# Alterations in peripheral levels of cytokines and associated inflammatory markers in acute and chronic stages of schizophrenia spectrum disorders: a systematic review and network meta-analysis

**DOI:** 10.1192/j.eurpsy.2023.1042

**Published:** 2023-07-19

**Authors:** S. Halstead, D. Siskind, M. Amft, E. Wagner, V. Yakimov, Z. Liu, K. Walder, N. Warren

**Affiliations:** 1 Logan Hospital, Queensland Health; 2 Faculty of Medicine, University of Queensland, Brisbane, Australia; 3 Department of Psychiatry and Psychotherapy, University Hospital, Ludwig Maximilian University of Munich, Munich, Germany; 4 The Institute for Mental and Physical Health and Clinical Translation, School of Medicine, Deakin University, Geelong, Australia

## Abstract

**Introduction:**

It has been previously identified that levels of peripheral inflammatory proteins, such as cytokines, are altered in people with schizophrenia spectrum disorders (SSD).

**Objectives:**

As there is considerable inconsistency in the literature with respect to how inflammatory profiles differ between acute and chronic stages of SSD, a systematic review and network meta-analysis was performed.

**Methods:**

Records from CINAHL, the Cochrane Central Register of Controlled Trials, EMBASE, PubMed, and PsycINFO were systematically searched from inception until 31 March 2022 for published studies that had measured levels of inflammatory proteins in cases of SSD and healthy controls. Pairwise and network meta-analyses were performed to determine whether there were significant differences in mean peripheral protein concentrations between acute SSD, chronic SSD, and healthy controls.

**Results:**

After application of the screening process, 215 articles were included for data-analysis. One group of markers were consistently elevated (p<0·05) in both acute and chronic SSD, relative to healthy controls; this group comprised interleukin (IL)-1β, IL-1 receptor antagonist (IL-1RA), soluble interleukin-2 receptor (sIL-2R), IL-6, IL-8, IL-10, tumor necrosis factor (TNF)-α, and high sensitivity C-reactive protein (hsCRP). A second group of markers were inconsistently altered between illness stages: IL-2 and interferon (IFN)-γ were significantly elevated (p<0·05) in acute SSD, whilst IL-4, IL-12 and IFN-γ were significantly decreased (p<0·05) in chronic SSD.

**Conclusions:**

These results indicate that a baseline level of inflammatory protein alteration occurs in SSD throughout the course of illness. This was evident from the group of markers that were consistently elevated in acute and chronic SSD (e.g., IL-6), representing possible trait markers. Moreover, superimposed immune activity may occur in acute SSD, given the group of possible state markers that were increased only in acute illness (e.g., IFN-γ). Further research is required to elucidate whether these peripheral changes are reflected within the central nervous system.

**Disclosure of Interest:**

None Declared

